# Practitioner's Bookshelf

**Published:** 1892-04-16

**Authors:** 


### PRACTITIONERS' BOOKSHELF.

This small volume* contains a great deal of useful and trustworthy information. It will be found of great service to the student who is preparing for his final examinations and has not sufficient time to read a longer treatise on public health. It can be confidently recommended to those who desire a brief and reliable account of the above subjects.

As a "cram" book† this is probably the least harmful of its kind. The questions asked are clear, concise, and to the point, and the answers, on the whole, are fairly complete without going into great detail. The information given is "up to date," and, so far as we have observed, reliable. The book may be found useful by those students who like to read a short summary of their subject before presenting themselves for examination.

* "Catechism Series. Public Health. Part V.—Medicine, Food, Burial, Water Closets, Disinfectants, Warming, Hospitals." (E. and S. Livingstone, Edinburgh). 1892.

† "Examination Questions in Practice of Medicine, with Answers. Part I.—General Diseases." (E. and S. Livingstone, Edinburgh). 1892.

